# Functional and anatomical connectivity predict brain stimulation’s mnemonic effects

**DOI:** 10.1093/cercor/bhad427

**Published:** 2023-12-01

**Authors:** Youssef Ezzyat, James E Kragel, Ethan A Solomon, Bradley C Lega, Joshua P Aronson, Barbara C Jobst, Robert E Gross, Michael R Sperling, Gregory A Worrell, Sameer A Sheth, Paul A Wanda, Daniel S Rizzuto, Michael J Kahana

**Affiliations:** Dept. of Psychology, Wesleyan University, Middletown, CT 06459, USA; Dept. of Neurology, University of Chicago, Chicago, IL 60637, USA; Perelman School of Medicine, University of Pennsylvania, Philadelphia, PA 19104, USA; Dept. of Neurosurgery, University of Texas Southwestern, Dallas, TX 75390, USA; Dept. of Neurosurgery, Beth Israel Deaconess Medical Center, Boston, MA 02215, USA; Dept. of Neurology, Dartmouth-Hitchcock Medical Center, Lebanon, NH 03756, USA; Dept. of Neurosurgery, Emory University Hospital, Atlanta, GA 30322, USA; Dept. of Neurology, Sidney Kimmel Medical College at Thomas Jefferson University, Philadelphia, PA 19107, USA; Department of Neurology, Mayo Clinic, Rochester, MN 55905, USA; Dept. of Neurosurgery, Baylor College of Medicine, Houston, TX 77030, USA; Dept. of Psychology, University of Pennsylvania, Philadelphia, PA 19104, USA; Dept. of Psychology, University of Pennsylvania, Philadelphia, PA 19104, USA; Dept. of Psychology, University of Pennsylvania, Philadelphia, PA 19104, USA

**Keywords:** brain stimulation, episodic memory, functional connectivity, white matter, intracranial EEG

## Abstract

Closed-loop direct brain stimulation is a promising tool for modulating neural activity and behavior. However, it remains unclear how to optimally target stimulation to modulate brain activity in particular brain networks that underlie particular cognitive functions. Here, we test the hypothesis that stimulation’s behavioral and physiological effects depend on the stimulation target’s anatomical and functional network properties. We delivered closed-loop stimulation as 47 neurosurgical patients studied and recalled word lists. Multivariate classifiers, trained to predict momentary lapses in memory function, triggered the stimulation of the lateral temporal cortex (LTC) during the study phase of the task. We found that LTC stimulation specifically improved memory when delivered to targets near white matter pathways. Memory improvement was largest for targets near white matter that also showed high functional connectivity to the brain’s memory network. These targets also reduced low-frequency activity in this network, an established marker of successful memory encoding. These data reveal how anatomical and functional networks mediate stimulation’s behavioral and physiological effects, provide further evidence that closed-loop LTC stimulation can improve episodic memory, and suggest a method for optimizing neuromodulation through improved stimulation targeting.

## Introduction

Direct electrical stimulation of the human brain can manipulate circuits underlying perception, cognition, and action (Scangos, Makhoul, et al. 2021; Siddiqi et al. 2022). Such stimulation has been used to treat network syndromes of brain dysfunction, suggesting that stimulation influences a broader network of brain regions beyond the stimulated location (Limousin et al. 1998; Mayberg et al. 2005; Deuschl et al. 2006; Lozano and Lipsman 2013; Geller et al. 2017; Jobst et al. 2017; Bouthour et al. 2019; Scangos, Khambhati, et al. 2021). Stimulation can also modulate behaviors, such as learning and memory, that depend on the coordinated activity of a network of brain regions (Voytek and Knight 2015; Keerativittayayut et al. 2018; Staresina and Wimber 2019; Mankin and Fried 2020; Das and Menon 2021).

Although increasingly used as a therapeutic and experimental tool, variability in outcomes poses a critical challenge, in part because stimulation’s mechanisms of action remain poorly understood. Theoretical accounts evolved from models of local disruption of pathological activity (Benabid et al. 2004) to modulation of the broader network of areas are connected to the stimulated location (McIntyre and Hahn 2010; Ashkan et al. 2017). If stimulation’s effects are best understood at the network level, perhaps variability in individual network structure can explain the variability in physiological and behavioral outcomes.

In support of this idea, applying stimulation to gray matter, the gray–white matter boundary, or specific white matter fibers, determines the spread of physiological effects through the network (Solomon et al. 2018; Paulk et al. 2022). Previous research has demonstrated different excitation thresholds for neural elements in white and gray matter (Nowak and Bullier 1998), which may explain variability in the spatial extent over which stimulation exerts its effects (Histed et al. 2009). Compared with gray matter stimulation, white matter stimulation leads to more broadly distributed excitation in downstream areas (Mohan et al. 2020; Crocker et al. 2021; Paulk et al. 2022). White matter pathways also constrain stimulation’s downstream functional effects (Lujan et al. 2013; Khambhati et al. 2019; Stiso et al. 2019). Behaviorally, stimulation of white matter has led to remission in depression (Mayberg et al. 2005), slowed cognitive decline in Alzheimer’s (Hamani et al. 2008; Lozano et al. 2016), and enhanced memory in epilepsy (Suthana et al. 2012; Titiz et al. 2017; Mankin et al. 2021). However, previous research has yet to show that variability in stimulation’s downstream effects depend on white vs. gray matter targeting in a way that predictably modulates episodic memory performance.

In addition to the brain’s anatomical architecture, research shows that functional architecture also mediates the spread and persistence of stimulation’s physiological effects (Keller et al. 2011, 2018; Fox et al. 2014, 2020). Previous work further suggests this relation to be frequency-specific. For example, stimulating targets in the medial temporal lobe leads to greater downstream changes in low-frequency (5–13 Hz) activity in brain regions that are strongly connected, at low-frequencies, to the stimulated site (Solomon et al. 2018). There are a variety of cognitive functions, including episodic memory, that have been linked to the modulation of low-frequency activity (Burke et al. 2013; Colgin 2013; Donoghue et al. 2020; Griffiths et al. 2021; Koster and Gruber 2022). Therefore, these physiological findings suggest that stimulating targets with strong low-frequency network connectivity could reliably modulate such behaviors, although this idea has yet to be tested. If such stimulation does affect broad low-frequency activity in a way that is related to behavior, it would be consistent with the notion that low-frequency activity coordinates function across a distributed neural network. Such coordination may be especially important for dynamic cognitive functions such as episodic memory (Watrous et al. 2013; Solomon et al. 2017) and would suggest that low-frequency activity may be a more effective target for modulation with stimulation than high-frequency activity (Fries 2009; Fell and Axmacher 2011; Harris and Gordon 2015).

We hypothesized that anatomical and functional characteristics of the stimulation target represent key variables that control the effect of stimulation on the brain’s memory network. We applied stimulation in closed-loop in 47 patients while they participated in an episodic memory task (free recall). We stimulated 57 targets located in the lateral temporal cortex (LTC), with the timing of stimulation determined by multivariate classification of neural activity during the encoding phase of the memory task. Activity in the LTC correlates with episodic memory performance (Ojemann et al. 1988; Kim 2011; Burke et al. 2014; Kragel et al. 2017), and stimulation studies targeting this area suggest that it may be an effective node for modulating the memory network (Bickford et al. 1958; Perrine et al. 1994; Moriarity et al. 2001; Flöel et al. 2008; Boggio et al. 2009; Curot et al. 2017; Ezzyat et al. 2018; Kucewicz et al. 2018). Using patient-specific data, we characterized each stimulation target based on its proximity to the nearest white matter pathway, as well as its low-frequency resting-state functional connectivity with the brain’s memory-encoding network. We found that closed-loop LTC stimulation improves memory performance relative to random stimulation, extending prior evidence that LTC stimulation modulates episodic memory (Ezzyat et al. 2018; Kucewicz et al. 2018). Furthermore, we reveal that stimulation target proximity to white matter and functional connectivity predict both stimulation’s effects on memory performance and changes in rhythmic low-frequency activity involved in successful memory encoding. These data suggest that in order to use stimulation effectively as a therapy for memory dysfunction, structural and functional characteristics of the stimulation target can be used to predictably modulate physiology and behavior (Ezzyat and Suthana In Press).

## Materials and methods

### Participants

Forty-seven patients undergoing intracranial electroencephalographic monitoring as part of clinical treatment for drug-resistant epilepsy were recruited to participate in this study. In total, *n* = 57 brain locations were stimulated: 38 patients were stimulated in one location, 8 patients were stimulated in two separate locations, and 1 patient was stimulated in three separate locations. Only one location was stimulated per session. Of the current dataset, data from 14 patients were included in an earlier publication (Ezzyat et al. 2018). All of the presently reported analyses and results are novel.

Data were collected as part of a multicenter project designed to assess the effects of electrical stimulation on memory-related brain function. Data were collected at the following centers: University of Texas Southwestern Medical Center (Dallas, TX), Dartmouth-Hitchcock Medical Center (Lebanon, NH), Thomas Jefferson University Hospital (Philadelphia, PA), Emory University Hospital (Atlanta, GA), Mayo Clinic (Rochester, MN), Hospital of the University of Pennsylvania (Philadelphia, PA), and Columbia University Medical Center (New York, NY). The research protocol was approved by the IRB at each hospital and informed consent was obtained from each participant. Electrophysiological data were collected from electrodes implanted subdurally (grid/strip configurations) on the cortical surface and/or electrodes within the brain parenchyma (depth electrodes). The clinical team has determined the placement of the electrodes based on the epileptogenic monitoring needs of the patient.

### Anatomical localization

Cortical surface regions were delineated on pre-implant whole brain volumetric *T*_1_-weighted MRI scans using Freesurfer (Fischl et al. 2004) according to the Desikan-Kiliany atlas (Desikan et al. 2006). Whole brain and high-resolution medial temporal lobe volumetric segmentation was also performed using the *T*_1_-weighted scan and a dedicated hippocampal coronal *T*_2_-weighted scan with Advanced Normalization Tools (ANTS) (Avants et al. 2008) and Automatic Segmentation of Hippocampal Subfields (ASHS) multiatlas segmentation methods (Yushkevich et al. 2015). Coordinates of the radiodense electrode contacts were derived from a postimplant CT and then registered with the MRI scans using ANTS. Subdural electrode coordinates were further mapped to the cortical surfaces using an energy minimization algorithm (Dykstra et al. 2012). Two neuro-radiologists reviewed cross-sectional images and surface renderings to confirm the output of the automated localization pipeline. Stimulation targets localized to the inferior, middle, or superior temporal gyri (left or right hemispheres) were classified as LTC. For region of interest (ROI) analyses, electrodes were assigned to regions using Freesurfer atlas labels (IFG: inferior frontal gyrus; MFG: middle frontal gyrus; SFG: superior frontal gyrus; MTLC: medial temporal lobe cortex; HIPP: hippocampus; ITG: inferior temporal gyrus; MTG: middle temporal gyrus; STG: superior temporal gyrus; IPC: inferior parietal cortex; SPC: superior parietal cortex; OC: occipital lobe).

### Verbal memory task

Across participants, data were collected from two behavioral tasks: standard delayed free recall and categorized delayed free recall. In both tasks, participants were instructed to study lists of words for a later memory test; no explicit encoding task was used. Lists were composed of 12 words presented in either English or Spanish, depending on the participant’s native language. In the standard free recall task, words were selected randomly from a pool of common nouns (https://memory.psych.upenn.edu/Word_Pools). In the categorized free recall task, the word pool was constructed from 25 semantic categories (e.g. fruit, furniture, and office supplies). Each list of 12 items in the categorized version of the task consisted of four words drawn from each of three categories. Overall, *n* = 19 participated in standard free recall only; *n* = 26 participated in categorized free recall only; and *n* = 2 participated in both free and categorized recall (in separate sessions).

Immediately following the final word in each list, participants performed a distractor task (to attenuate the recency effect in memory, length = 20 s) consisting of a series of arithmetic problems of the form *A* + *B* + *C* = ??, where *A*, *B*, and *C* were randomly chosen integers ranging from 1 to 9. Following the distractor task, participants were given 30 s to verbally recall as many words as possible from the list in any order; vocal responses were digitally recorded and later manually scored for analysis. Each session consisted of 25 lists of this encoding-distractor-recall procedure.

### E‌EG recording and analysis

Electrophysiological recording and stimulation was conducted using a variety of systems across the sites over which the project was conducted. Recording and stimulation equipment included clinical EEG systems (Nihon Kohden EEG-1200, Natus XLTek EMU 128, or Grass Aura-LTM64), equipment from Blackrock Microsystems (Salt Lake City, UT), as well as the External Neural Stimulator (ENS) Medtronic Inc. (Minneapolis, MN). Data were sampled at 500, 1,000, or 1,600 Hz (depending on the clinical site). During the sessions, a laptop recorded behavioral responses (vocalizations, key presses), synchronized to the recorded EEG via transmitted network packets.

Intracranial electrophysiological data were filtered to attenuate line noise (5-Hz band-stop fourth-order Butterworth, centered on 60 Hz). We referenced the data using a bipolar montage (Burke et al. 2013) by identifying all pairs of immediately adjacent contacts on every depth, strip, and grid and taking the difference between the signals recorded in each pair. The resulting bipolar timeseries was treated as a virtual electrode and used in all subsequent analysis. For the purposes of anatomical localization, we used the midpoint of the bipolar pair as the location for this virtual electrode. We used the same midpoint approach to localize stimulation targets and to measure stimulation target distance to white matter (see below).

### Multivariate classification of memory

We performed spectral decomposition (8 frequencies from 6 to 175 Hz, logarithmically spaced; Morlet wavelets; wave number = 5) for 1,366 ms epochs from 0 to 1,366 ms relative to word onset.

Mirrored buffers (length = 1,365 ms) were included before and after the interval of interest to avoid convolution edge effects. The resulting time–frequency data were then log-transformed, averaged over time, and *z*-scored within session and frequency band across word presentation events. For a subset of participants, we also performed the same spectral decomposition procedure on record-only data from the memory recall phase of each list. These data were then used in addition to the encoding data to train the classifier (Kragel et al. 2017). To do so, we computed spectral power for the 500-ms interval preceding a response vocalization, as well as during unsuccessful periods of memory search (the first 500 ms of any 2,500-ms interval in which no recall response was made). For both trial types (correct vocalizations and unsuccessful search periods), we further stipulated that no vocalization onsets occurred in the preceding 2,000 ms.

**Fig. 1:**
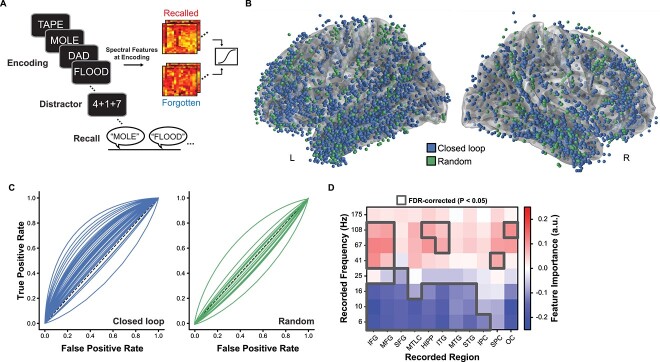
Multivariate decoding and classification of memory. A) Participants performed at least three sessions of the free recall task while being monitored with intracranial EEG. Multivariate classifiers were trained on whole-brain patterns of spectral activity to predict subsequently recalled vs. not recalled words. B) Recording electrode locations for all participants in the closed-loop and random groups rendered on the Freesurfer average brain. C) Eachparticipant’s multivariate classifier then served as their personalized model to trigger stimulation. Classifiers trained on record-only data generalized to the stimulation session(s) for the closed-loop group (*P* = 6.14 × 10^−7^) and outperformed classifiers for the random group (*P* = 2.73 × 10^−5^). D) An analysis of feature importance for classifiers from the closed-loop group showed that successful memory states were associated with decreases in low-frequency activity and increases in high-frequency activity.

Our closed-loop stimulation approach was based on using individualized memory classifiers to control the timing of stimulation in response to brain activity. Thus, after collecting at least three record-only sessions from an individual patient, we then used the data as input to a logistic regression classifier that would trigger closed-loop stimulation during the later stimulation session(s). To build the classifier, we used patterns of brain activity collected during record-only sessions and trained the classifier to discriminate words that were recalled vs. not recalled. The input features were spectral power at the eight analyzed frequencies × *N* electrodes (Fig. 1A). We used L2-penalization to prevent overfitting (Hastie et al. 2001) and set the penalty parameter (*C*) to 2.4 × 10^−4^ based on the optimal penalty parameter calculated across our large preexisting dataset of free-recall participants (Kragel et al. 2017; Ezzyat et al. 2018). We weighted the penalty parameter separately for each participant in inverse proportion to their number of recalled and not recalled words; this was done so that the model would learn equally from both classes (Hastie et al. 2001). Classification analyses were programmed using either the Matlab implementation of the LIBLINEAR library (Fan et al. 2008) or the Python library scikit-learn (Pedregosa et al. 2011).

For the Closed-loop group (34 participants, *n* = 40 stimulation targets), classifiers were trained using the true mapping of features (spectral power × electrodes) to recall outcomes. In contrast, for the Random group (13 participants, *n* = 17 stimulation targets), a technical error in labeling features during classifier training led to classifiers that were trained on permuted data, eliminating the true mapping between neural activity on each trial and recall outcomes. This provided a natural experiment for testing whether the closed-loop nature of stimulation can enhanced the efficacy of LTC stimulation.

To assess the importance of individual features to the classifier’s performance, we calculated the following forward model (Haufe et al. 2014):


$$ A=\frac{\varSigma \times \mathrm{w}}{\sigma_{\hat{\mathbf{y}}{\mkern6mu}}^2}, $$

where Σ_**x**_ is the data covariance matrix, **w** is the vector of feature weights from the trained classifier, and ${\sigma}_{\hat{\mathbf{y}}{\mkern6mu}}^2$ is the variance of the logit-transformed classifier outputs for all recalled/not recalled events ŷ. Positive values in *A* suggest a positive relation between power for a given feature and successful memory recall. We computed *A* separately for each participant (averaging features within anatomical ROIs based on the Freesurfer labels derived from anatomical localization of electrodes) before conducting across-participant statistical tests (Fig. 1D).

### Closed-loop stimulation

**Fig. 2:**
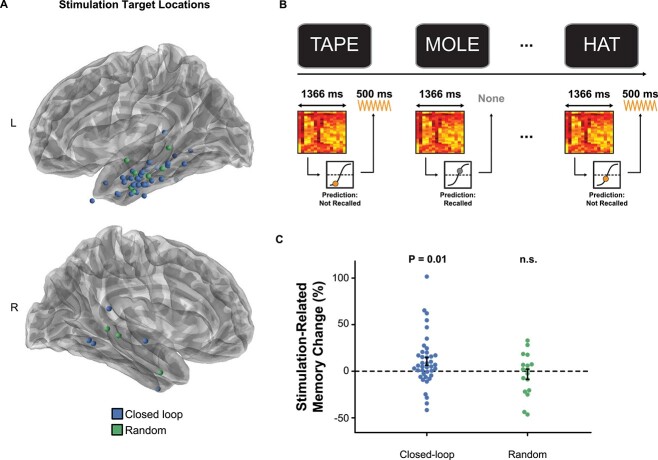
Closed-loop stimulation improves memory performance. A) Stimulation target locations for the closed-loop and random groups. B) Closed-loop stimulation strategy. C) Closed-loop LTC stimulation improved memory performance (*P* = 0.01), while random stimulation did not. Error bars in C reflect s.e.m.

At the start of each stimulation session, we determined the safe amplitude for stimulation using a mapping procedure in which stimulation was applied at 0.5 mA, while a neurologist monitored for afterdischarges. This procedure was repeated, increasing the amplitude in steps of 0.5 mA, up to a maximum of 1.5 mA for depth contacts and 3.5 mA for cortical surface contacts. These maximum amplitudes were chosen to be below the afterdischarge threshold and below accepted safety limits for charge density (Shannon 1992). For each stimulation session, we passed electrical current through a single pair of adjacent electrode contacts. The locations of implanted electrodes were determined strictly by the monitoring needs of the clinicians (recording sites depicted in Fig. 1B). We therefore used a combination of anatomical and functional information to select stimulation sites, prioritizing (if available) targets in the middle temporal gyrus (stimulation targets depicted in Fig. 2A). This choice was guided by prior work identifying the middle temporal gyrus as an effective target for modulating memory with stimulation (Ezzyat et al. 2018; Kucewicz et al. 2018). Stimulation was delivered using charge-balanced biphasic rectangular pulses (pulse width = 300 μs) at either 50-, 100-, or 200-Hz frequency (a single frequency was chosen for each subject) and was applied for 500 ms in response to classifier-detected poor memory states (see below). Participants performed one practice list followed by 25 task lists: lists 1–3 were used as a baseline for normalizing the classifier; lists 4–25 consisted of 11 lists each of Stim and NoStim conditions, randomly interleaved. NoStim lists were identically structured to Stim lists, except that stimulation was never delivered in response to classifier output.

To determine (in actuality) how well the classifier predicted recalled and forgotten words in a given participant’s stimulation session, we again used AUC. We used the true classifier outputs and true recall outcomes from the NoStim lists to calculate the classifier generalization AUC for the stimulation sessions. To generate the corresponding receiver operating characteristic curves for visualization (Fig. 2C), we modeled the classifier outputs for recalled and not recalled words using signal detection theory (Wixted 2007). We did this by using the classifier outputs to estimate the mean and variance of hypothetical (normal) distributions of memory strength for recalled and not recalled words. We then generated a curve relating true and false positive rates by varying the assumed decision criterion (Wixted 2007).

### Analysis of memory performance

All participants completed at least three sessions of the record-only task (for purposes of classifier training) and at least one session of the stimulation task. For the stimulation session(s), we calculated stimulation’s effect on recall performance as follows:


$$ \varDelta =\frac{R_S-{R}_{NS}}{R_{NS}}\times 100, $$

where *R_S_* is the average recall for stimulated lists and *R_NS_* is the average recall for nonstimulated lists. Because the first three lists of every stimulation session were always nonstimulated (used for normalization of the classifier input features for that session), we excluded these lists from the calculation of *R_NS_* to avoid introducing a temporal order confound (Ezzyat et al. 2018). All participants were required to demonstrate a minimum *R_NS_* = 8.33% (1 out of 12 words per list) for inclusion in the sample.

### Calculation of stimulation target distance to white matter

Using Freesurfer to segment patients’ *T*_1_ MRI scan, we identified white-matter vertex locations, and then we calculated the distance between the stimulation location (midpoint of the bipolar pair) and the nearest white matter vertex. These distances were then split into thirds in order to categorize stimulation sites as Near, Mid, or Far relative to the nearest white matter (Solomon et al. 2018; Mohan et al. 2020).

### Calculation of stimulation target node strength

We adapted a previously reported method for calculating the resting-state functional connectivity between channels using the MNE-Python software package (Gramfort et al. 2014; Solomon et al. 2018). We extracted data from nontask periods of the record-only sessions of each patient and used the data to calculate the coherence between each pair of bipolar channels in the patient’s montage. The coherence (*C_xy_*) between two signals is the normalized cross-spectral density. This measure reflects the consistency of phase differences between signals at two electrodes, weighted by the correlated change in spectral power at both sites


$$ {C}_{xy}=\frac{S_{xy}}{S_{xx}{S}_{yy}}, $$

where *S_xy_* is the cross-spectral density between signals at electrodes *x* and *y*; *S_xx_* and *S_yy_* are the autospectral densities at each electrode. We used the multitaper method to estimate spectral density (Bronez 1992). We used a time-bandwidth product of 4 and a maximum of 8 tapers (tapers with spectral energy <0.9 were removed), computing coherence for frequencies between 5 and 13 Hz. We computed interelectrode coherence within nonoverlapping 1-s windows of data collected during a 10-s baseline (countdown) period that occurred at the start of each word list. The resulting coherence values between each pair of electrodes were then regressed on the Euclidean distance between each pair of electrodes, to account for the correlation between interelectrode coherence and distance (Solomon et al. 2018). This distance-residualized measure of coherence was then used in the node-strength calculation. We repeated this entire procedure for calculating high-frequency functional connectivity in the 45–90 Hz range.

### Analysis of physiological effects of stimulation

To assess the effect of LTC stimulation on neural activity, we analyzed recording channels (i.e. those that were not stimulated) and we compared stimulation-evoked spectral power separately at low and high frequencies. We first excluded electrodes exhibiting nonphysiological poststimulation artifacts (such as amplifier saturation/relaxation) using three different measures of the EEG timeseries before and after stimulation. We compared intervals before and after stimulation for changes in variance using an *F*-test and for changes in signal amplitude using a *t*-test. We additionally fit a polynomial function to the timeseries before and after each stimulation event and used a *t*-test to compare the resulting parameter estimates for the quadratic term. We calculated these three measures using the signal from −400 to −100 ms relative to stimulation onset and from 100 to 400 ms relative to stimulation offset. In order to select statistical thresholds for each measure, we conducted the same analysis on each participant’s record-only data. We then selected *P*-value thresholds associated with a 5% detection rate in the record-only data (i.e. false positives). Any channel that was significant on any of the three measures was excluded from analysis.

To measure stimulation’s effect on low-frequency power, we extracted spectral power from −600 to −100 ms relative to stimulation onset and from 100 to 600 ms relative to stimulation offset. We used Morlet wavelets (wave number = 5) to estimate spectral power for the same set of frequencies used to train the classifier with buffers to eliminate edge artifacts. The resulting spectral power estimates were then *z*-scored within each frequency, separately for each session. We then averaged power within each frequency across the time dimension for each prestimulation period and for each matched poststimulation period. We then subtracted the prestimulation data from the poststimulation data to yield a distribution of change in spectral power for each electrode.

We compared the distribution of power changes for stimulation events to the power changes from matched intervals on NoStim lists. To do so, we calculated spectral power using identical parameters. However, because there were no actual stimulation events in NoStim lists, we generated a synthetic distribution of stimulation onset times by extracting the lag (in milliseconds) between each word onset and stimulation event in Stim lists, and sampling randomly from that distribution of onset times to determine when to extract data relative to word onset events in NoStim lists.

Finally, we used an independent samples *t*-test to compare the distribution of Stim list power differences to the distribution of NoStim list power differences within each electrode. The resulting distribution of *t*-statistics was then averaged across electrodes to estimate the stimulation-evoked change in power (Fig. 5A). We then averaged these values separately within clusters of low and high frequencies that significantly predicted memory performance (based on classifier feature importance, Fig. 1D).

### Statistics

**Fig. 3:**
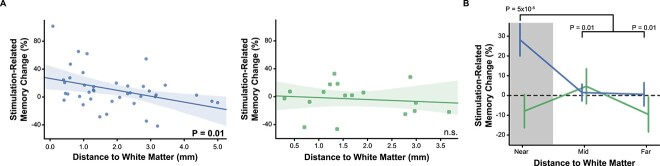
Closed-loop stimulation near white matter enhances memory. A) For the closed-loop group, stimulation’s effect on memory depended on the target distance from the nearest white matter [left, *P* = 0.007]. The correlation was not significant for the random group [right, *P* = 0.52]. B) Closed-loop LTC stimulation improved memory performance for targets located nearest to white matter (*P* = 0.005). There was no effect for the random group (*P* = 0.62). Error regions in A reflect the standard error of the estimate. Error bars in B reflect s.e.m.

Data are presented as mean ± standard error of the mean; scatterplots show the standard error of the estimate. All statistical comparisons were conducted as two-tailed tests. Nonparametric tests (e.g. Mann–Whitney; Wilcoxon signed rank) were used for nonnormally distributed variables (e.g. white matter distance, Fig. 3A). To account for the fact that some participants were stimulated at more than one target (always in separate sessions), we used linear mixed effects models to assess the effect of stimulation on memory and differences between the Closed-loop and Random groups. The models assumed separate intercepts and slopes for each participant. We also used linear mixed effects models in analyzing the effects of white matter distance on memory; low- and high-frequency memory network node strength; and the effect of node strength on stimulation-evoked physiology. Data distributions were visually inspected or assumed to be normal for parametric tests.

## Results

### Multivariate classifiers identify memory lapses

Our stimulation strategy sought to intercept and rescue periods of poor memory encoding. To do so, we trained participant-specific multivariate classifiers to discriminate patterns of neural activity during record-only sessions of free recall (Fig. 1A). For the Closed-loop group (*n* = 40), classifiers were trained using the true mapping of features (spectral power × electrodes) to recall performance; for the Random group (*n* = 17), due to a technical error in labeling features (see Materials and methods), classifiers were trained on permuted features. The recording electrode locations for the Closed-loop and Random groups appear as spheres in Fig. 1B. After training the classifiers on record-only data, we used them in later (independent) sessions to identify poor memory states for targeting with stimulation.

Our first question was how well the classifiers predicted memory outcomes during the stimulation sessions (i.e. out-of-sample generalization). To answer this question, we used data from NoStim lists in which we obtained classifier predictions about the probability of recall for each word but did not use these predictions to trigger stimulation (see Materials and methods). Using area under the receiver operating characteristic curve as an index of classification accuracy, we found that classifiers for the Closed-loop reliably exceeded chance performance [Mean AUC = 0.62 (chance AUC = 0.50), Wilcoxon signed rank test *P* = 5.73 × 10^−7^]. Closed-loop classifiers also outperformed classifiers for the Random group (Mann–Whitney *U* = 586.0, *P* = 1.85 × 10^−5^). As expected, Random classifiers did not exceed chance (Mean AUC = 0.49, Wilcoxon signed rank test *P* = 0.55; Fig. 1C).

To understand what features the classifier used to discriminate good vs. poor memory encoding states, we used a forward model for each participant to derive importance estimates for each feature (Haufe et al. 2014). We averaged the feature importance values within a set of ROIs separately for each classifier frequency. Across participants, classifiers predicted successful memory encoding based on increased high-frequency activity (especially in frontal, lateral temporal, and medial temporal lobe areas) and decreased low-frequency activity across much of the recorded cortex and subcortex (Fig. 1D). This pattern, which we refer to as the spectral tilt, has been observed in previous studies to be a biomarker of successful episodic memory encoding and retrieval (Burke et al. 2014; Long et al. 2014; Ezzyat et al. 2017).

### Closed-loop LTC stimulation improves memory

Having established that classifiers in the Closed-loop group reliably discriminate memory encoding states, we next asked if we could increase memory performance via stimulation of the LTC (Fig. 2A). Our stimulation strategy was based on detecting poor memory encoding states and intercepting them with stimulation (Fig. 2B). For the Closed-loop and Random groups, we compared recall performance for lists in which we delivered stimulation (Stim lists) vs. identically structured lists in which we did not stimulate (NoStim lists, as described above). In the Closed-loop group, recall was higher on Stim lists compared with NoStim lists (∆ = 10.4% ± 4.2; *z* = 2.5, *P* = 0.01, Fig. 2C), suggesting that intercepting poor memory encoding states with LTC stimulation enhanced recall. In contrast, there was no difference in memory performance for the Random group (∆ = −3.4% ± 6.6; *z* = −0.52, *P* = 0.60). There was a trend for greater memory enhancement for the Closed-loop compared with the Random group (*z* = 1.77, *P* = 0.08). These findings are the first to compare closed-loop LTC stimulation with a random/open-loop stimulation control and are consistent with previous studies showing memory enhancement via LTC stimulation (Ezzyat et al. 2018; Kucewicz et al. 2018; Kahana et al. 2023).

### White matter proximity mediates stimulation’s effect on memory

Motivated by physiological studies of electrical stimulation’s effects on downstream targets (Keller et al. 2018; Solomon et al. 2018; Mohan et al. 2020), we asked whether stimulating close to white matter tracts would produce greater positive or negative effects on memory. If so, this would suggest that the brain’s anatomical network structure plays a key role in determining how effectively stimulation can modulate cognitive function (Stiso et al. 2019; Crocker et al. 2021). To answer this question, we examined how stimulation’s effect on memory performance varied as a function of the stimulation target’s proximity to white matter. For the Closed-loop group, lower distance to white matter predicted greater stimulation-related memory improvement (*z* = 3.51, *P* = 0.01; Fig. 3A). In the random stimulation group, we neither expected nor observed a correlation between white matter distance and the memory effect (*P* = 0.66). There was no difference between the distances to white matter for the Closed-loop and Random groups (*P* = 0.65) and the median distance was in fact numerically greater for the Closed loop (1.58 mm) compared with the Random group (1.39 mm). This suggests that distance to white matter alone does not explain the finding of improved memory in the Closed-loop group. Instead, proximity to white matter appears to enhance the effectiveness of closed-loop stimulation.

To further test this idea, we divided stimulation targets into terciles and asked whether stimulation near white matter was particularly effective in modulating memory performance.

Indeed, Closed-loop stimulation targets near white matter enhanced memory performance on Stim lists compared with NoStim lists (Near: *M* = 28.3% ± 7.0%, *z* = 4.06, *P* = 5 × 10^−5^). This memory improvement was larger than for Closed-loop stimulation targets further away from white matter (Mid: *M* = 1.5% ± 7.4%, *z* = 2.59, *P* = 0.01; Far: *M* = 0.6% ± 7.4%, *z* = 2.61, *P* = 0.009). Closed-loop stimulation near white matter also significantly outperformed the Random stimulation near white matter group (Random *M* = −7.9% ± 13.3%, *z* = 2.31, *P* = 0.02, Fig. 3B). As expected, the Random group did not show improved memory (Stim vs. NoStim within-participant) in any white matter distance bin (all *P* > 0.32). These data suggest that stimulating near white matter leads to greater modulation of memory, and extend previous work that linked white matter proximity to stimulation’s effect on electrophysiology (Keller et al. 2018; Solomon et al. 2018; Stiso et al. 2019; Mohan et al. 2020; Crocker et al. 2021).

### Stimulation target functional connectivity predicts the change in memory

**Fig. 4:**
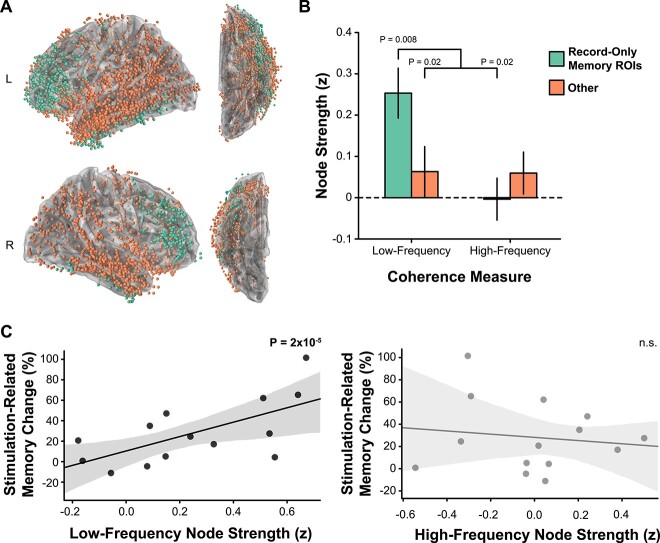
Stimulation target functional connectivity. A) We assigned each patient’s record-only electrodes to two ROIs based on whether the electrode was located in a region that showed a memory-related spectral tilt or not (other). B) Low-frequency connectivity was higher between the stimulation target and electrodes in classifier-defined memory regions, compared with electrodes in other regions (*P* = 0.02) and compared with high-frequency network connectivity (*P* = 0.02). In contrast, there was no difference in stimulation target high-frequency network connectivity. C) For closed-loop targets nearest to white matter, there was a significant correlation between stimulation target low-frequency connectivity and stimulation’s effect on memory [β = 0.69, *P* = 2 × 10^−5^]. There was no effect for high-frequency connectivity. Error bars reflect s.e.m. Error regions reflect the standard error of the estimate.

We next asked why closed-loop stimulation delivered near white matter reliably modulated memory function. One possibility is that stimulating near white matter allows more reliable and direct access to the broader memory network connected to the stimulated location (Solomon et al. 2018; Khambhati et al. 2019; Stiso et al. 2019; Mohan et al. 2020). We therefore measured functional connectivity between the brain’s memory encoding network and the stimulation targets located near white matter. Critically, we constructed separate measurements of connectivity at low (5–13 Hz) and high frequencies (45–90 Hz) by calculating coherence using participant-specific resting-state data (see Materials and methods). Then, to isolate the brain’s memory encoding network, we identified all electrodes that were in brain regions that showed a spectral tilt that predicted memory success during the task, assessed using classifier feature importance Fig. 4A. We then compared stimulation target connectivity to electrodes In vs. Out of the memory network, for both low- and high-frequency coherence (referred to as Node Strength). Stimulation targets showed stronger low-frequency connectivity to electrodes in the memory network than to electrodes outside of the memory network [*z* = 2.31, *P* = 0.02, Fig. 4B]. For memory network electrodes, low-frequency connectivity was also higher than high-frequency connectivity [*z* = 2.39, *P* = 0.02]. In contrast, stimulation targets showed equivalent high-frequency connectivity In vs. Out of the memory network [*P* = 0.49, Fig. 4B].

Although stimulation targets near white matter showed greater overall low-frequency connectivity with memory-predicting brain areas, this finding leaves open the question of whether variability in connectivity strength with the memory network predicts variability stimulation’s effect on memory. To answer this question, we correlated low-frequency node strength with stimulation-related memory change. We found that low-frequency node strength predicted closed-loop stimulation’s effect on memory (β = 0.69 ± 0.16, *P* = 2 × 10^−5^, Fig. 4C), while high-frequency node strength did not (*P* = 0.56). The difference in correlation for low- vs. high-frequency node strength was also significant (two-tailed permutation test *P* = 0.03). For all other targets that were further from white matter, there was no relation between node strength and stimulation-related memory change (all *P* > 0.19).

### Functional connectivity mediates stimulation’s effect on downstream physiology

**Fig. 5:**
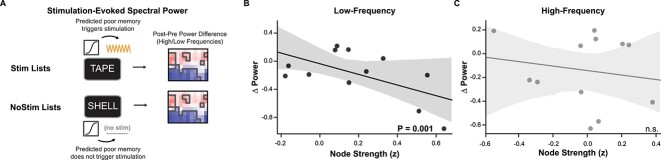
Memory network connectivity predicts physiology. A) Schematic of the analysis of stimulation-evoked physiology. B) For stimulation targets near white matter, low-frequency functional connectivity predicted the stimulation-evoked change in low-frequency power (*P* = 0.02). C) High-frequency network connectivity did not predict stimulation’s effect on high-frequency activity.

The preceding results indicate that low-frequency functional connectivity to the memory network predicts stimulation effects on memory. Our final question was whether low-frequency connectivity also predicts stimulation’s physiological effects across the memory network. To test this prediction, we again examined Closed-loop stimulation targets near white matter and correlated each stimulation target’s connectivity to the memory network with the stimulation-evoked spectral power in this network (Fig. 5A). Two participants’ data were excluded due to excessive stimulation artifact on the recording channels. In the remaining participants, we found that stimulation-target functional connectivity predicted stimulation-related changes in low-frequency power (β = −0.72 ± 0.23, *P* = 0.001, Fig. 5B). The correlation was not significant when using high-frequency connectivity and evoked power (*P* = 0.83, Fig. 5C).

## Discussion

Direct electrical stimulation has emerged as a powerful tool for manipulating neural activity. The present study evaluated the hypothesis that network properties of a stimulated brain location predict stimulation’s effects on both memory and network physiology. Prior studies suggest that white matter pathways mediate stimulation’s network-level physiological effects (Solomon et al. 2018; Khambhati et al. 2019; Stiso et al. 2019; Mohan et al. 2020; Paulk et al. 2022). Other studies demonstrate that measures of structural and functional connectivity predict stimulation’s effects on downstream targets (Keller et al. 2011; Solomon et al. 2018; Fox et al. 2020). However, none have simultaneously linked structural/functional connectivity with both (i) a reliable improvement over baseline cognitive functioning and (ii) concomitant changes in neurophysiology that explain the behavioral effect. To directly address these questions, we asked whether white-matter proximity and functional connectivity underlie the degree to which stimulation of LTC produces improvements or impairments of memory, alongside changes in oscillatory signatures of mnemonic function.

We found that closed-loop stimulation of LTC reliably improved memory on stimulated vs. nonstimulated lists. Consistent with the hypothesis that white-matter pathways convey the effects of stimulation to the broader memory network, we found the benefits of closed-loop LTC stimulation to arise principally from stimulating in, or near, white matter pathways. For the electrodes nearest to white matter, stimulation yielded a 28% increase in recall performance, whereas we failed to observe any reliable increase when delivering stimulation far from these pathways (1%). In a subgroup of subjects who received randomly timed stimulation in LTC targets, we failed to observe any improvement in memory performance.

To evaluate how stimulation–target functional connectivity mediates stimulation’s behavioral and physiological effects, we analyzed participant-specific large-scale neural recordings obtained during prior record-only sessions. Prior studies have shown that brain networks become coherent at low-frequencies during successful memory encoding and retrieval (Solomon et al. 2017; Kragel, Ezzyat, et al. 2021), so we used low-frequency coherence to measure the network node strength of each stimulation target. We then asked if greater node strength between LTC stimulation targets and downstream memory-predicting areas resulted in greater effects of stimulation on memory performance. Consistent with this hypothesis, we found a strong positive correlation (β = 0.69, Fig. 4C) between low-frequency connectivity and stimulation-related memory improvement. Finally, LTC stimulation engaged low-frequency activity across a broader brain network in a way that matched the network position of the stimulated location (Fig. 5).

Our data highlight how precise targeting improves stimulation efficacy by showing that delivering stimulation near LTC white-matter leads to greater stimulation-related memory gains (Fig. 3C). By linking low-frequency network connectivity with physiological and behavioral outcomes, our study also points to a neural mechanism for modulating memory with stimulation. This result extends earlier work that demonstrated the potential to modulate episodic memory by targeting LTC with stimulation (Ezzyat et al. 2018; Kucewicz et al. 2018). Directly comparing closed-loop and open-loop stimulation strategies in the same study helps to establish a causal role for the closed-loop approach (Ezzyat and Rizzuto 2018; Hampson et al. 2018). Finally, our data from 57 stimulation targets (across 47 patients) also represent a substantial increase compared with sample sizes described in related prior studies (Ezzyat et al. 2018; Hampson et al. 2018).

Prior work has linked successful memory function with theta power and coherence (Klimesch et al. 1997; Guderian and Düzel 2005; Osipova et al. 2006; Burke et al. 2013; Staudigl and Hanslmayr 2013; Solomon et al. 2017; Griffiths et al. 2019; Herweg et al. 2020; Ter Wal et al. 2021; Kragel, Schuele, et al. 2021). Here, we investigated this physiological correlate of memory function by testing how memory-modulating LTC stimulation affects low-frequency physiology. We found that stimulation’s effect on low-frequency activity depends on the low-frequency functional connectivity of the stimulation target. This suggests that identifying strong functional connections can produce stronger modulation of low-frequency activity within the memory network. Furthermore, we found that stimulation that modulated low-frequency activity also modulated memory performance.

Several prior studies found potential therapeutic benefits of closed-loop stimulation triggered by decoding of intracranial brain recordings (Ezzyat et al. 2018; Hampson et al. 2018; Scangos, Khambhati, et al. 2021; Kahana et al. 2023). However, with some important exceptions (Hampson et al. 2018), this work has lacked an open-loop or random stimulation control condition, leaving open the question of what specific role the closed-loop nature of stimulation played in its therapeutic effects. Here, we compared the effects of closed-loop stimulation with a random stimulation condition. Closed-loop participants received stimulation only for those items predicted to be forgotten. Participants in the Random group followed the same protocol, but using classifiers trained on permuted data, resulting in stimulation being applied without regard to predicted memory success. This led to reliable memory improvement for the Closed-loop group and none for the Random group, despite following an otherwise identical protocol (Fig. 2C).

We found that closed-loop stimulation improved memory the most when it was delivered to LTC targets in or near white matter. This finding builds on a growing literature that indicates that stimulation is most effective when it is delivered in or near white matter pathways (Solomon et al. 2018; Khambhati et al. 2019; Stiso et al. 2019;Mohan et al. 2020 ; Paulk et al. 2022). One explanation for this phenomenon is that only stimulation of white matter pathways successfully engages broader brain networks, perhaps via oscillatory synchronization. In contrast, gray matter stimulation tends to cause more local effects (Mohan et al. 2020; Paulk et al. 2022). Though purely local effects may sometimes be desirable, the key cognitive and pathophysiological processes of greatest interest to neuroscientists tend to involve multiple interconnected brain regions.

Among its many applications for modulating cognition and behavior (Sreekumar et al. 2017; Fox et al. 2020; Siddiqi et al. 2022), a number of recent studies have evaluated stimulation’s potential for enhancing episodic memory (Lee et al. 2013; Sankar et al. 2014; Suthana and Fried 2014; Curot et al. 2017; Mankin and Fried 2020). While our study investigated numerous stimulation targets within the LTC, future work should compare stimulation of this region to other brain areas within the broader episodic memory network. Recent work suggests that stimulating white matter pathways in the medial temporal lobe, for example, can also improve memory (Suthana et al. 2012; Titiz et al. 2017; Mankin et al. 2021). However, these previous studies used visual and/or spatial memoranda, while the present study focused on encoding and retrieval of verbal material. Thus, future research should compare stimulation to the lateral and medial temporal lobes, to determine whether stimulation target location interacts with the modality of the to-be-remembered information. This could contribute to other work that has used stimulation to study the component processes that contribute to successful episodic memory (El et al. 2019).

We delivered stimulation using macroelectrodes, consistent with its clinical applications (Sun et al. 2008; Morrell 2011; Krauss et al. 2021). Macroelectrode stimulation alters local activity at the spatial scale of the distance between the anode and cathode (approximately 1 cm) but can also alter more distant regions. Because memory relies on a broad network of cortical and subcortical regions, including the hippocampus (Kim 2011; Keerativittayayut et al. 2018), stimulating a broader network may be necessary to impact cognitive function. On the other hand, memory also relies on the recapitulation of specific patterns of neuronal activity, especially within the hippocampus (Foster 2017; Staresina and Wimber 2019). Thus, other work has stimulated through microelectrodes to mimic and reinstate memory-related hippocampal activity using a model-based closed-loop approach (Hampson et al. 2013, Deadwyler et al. 2017, Hampson et al. 2018). An avenue for future work could use macroelectrode stimulation in a similar vein, by triggering stimulation at multiple macroelectrode contacts in order to synchronize a particular spatiotemporal pattern of activity across key memory-related regions (Kim et al. 2016, 2018).

In relating low-frequency network connectivity, physiology, and behavior, our study contributes to the methodological development for invasive stimulation (Cagnan et al. 2019; Krauss et al. 2021) that illuminates the critical role of low-frequency networks in cognition (Voytek and Knight 2015). In addition, the present study also suggests that other methods that manipulate low-frequency activity could be leveraged to modulate neural and cognitive function. Several recent studies using noninvasive methods have leveraged low-frequency theta-patterned stimulation to modulate episodic and working memory (Nilakantan et al. 2017; Tambini et al. 2018; Warren et al. 2019; Hermiller et al. 2020; Grover et al. 2022). Such low-frequency stimulation modulates electrophysiology perhaps by entraining low-frequency oscillations that are associated with cognitive function (Reinhart et al. 2017; Hanslmayr et al. 2019; Reinhart and Nguyen 2019; Solomon et al. 2021).

There are some limitations to the current work that emerge from trade-offs associated with our approach. We attempted to construct the largest existing dataset examining invasive closed-loop stimulation for memory modulation. In so doing, although most participants were stimulated at a single frequency (200 Hz), we included in our analysis participants who underwent stimulation at other stimulation frequencies. A critical question for future research will be to directly compare stimulation at different frequencies within-participant (Mohan et al. 2020). In addition, we carried out our comparisons of (i) closed-loop vs. random stimulation and (ii) white matter distance as between-participants tests. Future work should therefore address the effectiveness of closed-loop stimulation and white matter targeting within-participant, by focusing resources on collecting data within-participant. In the case of invasive stimulation, however, this approach presents its own challenges in terms of patients’ clinical priorities.

In summary, our demonstration of improved memory with closed-loop stimulation supports the idea that memory function is dynamic, and that closed-loop algorithms that account for moment-to-moment variability in the brain’s memory state can selectively deliver stimulation only when it is needed. The present study also links closed-loop stimulation efficacy to white matter targeting, brain-wide evoked physiology, and changes in episodic memory performance. The findings suggest future strategies for using the functional and anatomical network profile of putative stimulation targets to optimize downstream changes in oscillatory activity and cognition.

## Data Availability

Upon publication, all deidentified raw data and analysis code may be downloaded at http://memory.psych.upenn.edu/Electrophysiological_Data.

## References

[ref1] Ashkan K , Rogers P, Bergman H, Ughratdar I. Insights into the mechanisms of deep brain stimulation. Nat Rev Neurol. 2017:13(9):548–554. 28752857 10.1038/nrneurol.2017.105

[ref2] Avants BB , Epstein CL, Grossman M, Gee JC. Symmetric diffeomorphic image registration with cross-correlation: evaluating automated labeling of elderly and neurodegenerative brain. Med Image Anal. 2008:12(1):26–41. 17659998 10.1016/j.media.2007.06.004 PMC2276735

[ref3] Benabid A , Grand S, Benazzouz A, Pollack P, Krack P, Koudsie A, Chabardes S, Fraix V, Limousin P, Pinto S, et al. Deep brain stimulation for movement disorders. In: Winn H, editors. Youman’s neurosurgical surgery. Vol. 3. 5th ed. Philadelphia (PA): Saunders; 2004. pp. 2803–2827

[ref4] Bickford RG , Mulder DW, Dodge HW Jr, Svien HJ, Rome HP. Changes in memory function produced by electrical stimulation of the temporal lobe in man. Res Publ Assoc Res Nerv Ment Dis. 1958:36:227–243. 13527786

[ref5] Boggio P , Fregni F, Valasek C, Ellwood S, Chi R, Gallate J, Pascual-Leone A, Snyder A. Temporal lobe cortical electrical stimulation during the encoding and retrieval phase reduces false memories. PLoS One. 2009:4(3):e4959. 10.1371/journal.pone.0004959 PMC2655647 19319182

[ref6] Bouthour W , Mégevand P, Donoghue J, Lüscher C, Birbaumer N, Krack P. Biomarkers for closed-loop deep brain stimulation in parkinson disease and beyond. Nature reviews. Neurology. 2019:15(6):343–352. 30936569 10.1038/s41582-019-0166-4

[ref7] Bronez TP . On the performance advantage of multitaper spectral analysis. IEEE Trans Signal Process. 1992:40(12):2941–2946.

[ref8] Burke JF , Zaghloul KA, Jacobs J, Williams RB, Sperling MR, Sharan AD, Kahana MJ. Synchronous and asynchronous theta and gamma activity during episodic memory formation. J Neurosci. 2013:33(1):292–304. 23283342 10.1523/JNEUROSCI.2057-12.2013 PMC3711714

[ref9] Burke JF , Sharan AD, Sperling MR, Ramayya AG, Evans JJ, Healey MK, Beck EN, Davis KA, Lucas TH, Kahana MJ. Theta and high–frequency activity mark spontaneous recall of episodic memories. J Neurosci. 2014:34(34):11355–11365. 25143616 10.1523/JNEUROSCI.2654-13.2014 PMC4138344

[ref10] Cagnan H , Denison T, McIntyre C, Brown P. Emerging technologies for improved deep brain stimulation. Nat Biotechnol. 2019:37(9):1024–1033. 31477926 10.1038/s41587-019-0244-6 PMC6877347

[ref11] Colgin LL . Mechanisms and functions of theta rhythms. Annu Rev Neurosci. 2013:36(1):295–312. 23724998 10.1146/annurev-neuro-062012-170330

[ref12] Crocker B , Ostrowski L, Williams ZM, Dougherty DD, Eskandar EN, Widge AS, Chu CJ, Cash SS, Paulk AC. Local and distant responses to single pulse electrical stimulation reflect different forms of connectivity. NeuroImage. 2021:237:118094. 33940142 10.1016/j.neuroimage.2021.118094 PMC12424142

[ref13] Curot J , Busigny T, Valton L, Denuelle M, Vignal JP, Maillard L, Chauvel P, Pariente J, Trebuchon A, Bartolomei F, et al. Memory scrutinized through electrical brain stimulation: a review of 80 years of experiential phenomena. Neurosci Biobehav Rev. 2017:78:161–177. 28445741 10.1016/j.neubiorev.2017.04.018

[ref14] Das A , Menon V. Asymmetric frequency-specific feedforward and feedback information flow between hippocampus and prefrontal cortex during verbal memory encoding and recall. J Neurosci. 2021:41(40):8427–8440. 34433632 10.1523/JNEUROSCI.0802-21.2021 PMC8496199

[ref15] Deadwyler SA , Hampson RE, Song D, Opris I, Gerhardt GA, Marmarelis VZ, Berger TW. A cognitive prosthesis for memory facilitation by closed-loop functional ensemble stimulation of hippocampal neurons in primate brain. Exp Neurol. 2017:287(Pt 4):452–460. 27233622 10.1016/j.expneurol.2016.05.031 PMC5633045

[ref16] Desikan R , Segonne B, Fischl B, Quinn B, Dickerson B, Blacker D, Buckner RL, Dale A, Maguire A, Hyman B, et al. An automated labeling system for subdividing the human cerebral cortex on MRI scans into gyral based regions of interest. NeuroImage. 2006:31(3):968–980. 16530430 10.1016/j.neuroimage.2006.01.021

[ref17] Deuschl G , Schade-Brittinger C, Krack P, Volkmann J, Schäfer H, Bo¨tzel K, Daniels C, Deutschländer A, Dillmann U, Eisner W, et al. A randomized trial of deep-brain stimulation for Parkinson’s disease. N Engl J Med. 2006:355(9):896–908. 16943402 10.1056/NEJMoa060281

[ref18] Donoghue T , Haller M, Peterson EJ, Varma P, Sebastian P, Gao R, Noto T, Lara AH, Wallis JD, Knight RT, et al. Parameterizing neural power spectra into periodic and aperiodic components. Nat Neurosci. 2020:23(12):1655–1665. 33230329 10.1038/s41593-020-00744-x PMC8106550

[ref19] Dykstra AR , Chan AM, Quinn BT, Zepeda R, Keller CJ, Cormier J, Madsen JR, Eskandar EN, Cash SS. Individualized localization and cortical surface-based registration of intracranial electrodes. NeuroImage. 2012:59(4):3563–3570. 22155045 10.1016/j.neuroimage.2011.11.046 PMC3288767

[ref20] El-Kalliny MM , Wittig JH Jr, Sheehan TC, Sreekumar V, Inati SK, Zaghloul KA. Changing temporal context in human temporal lobe promotes memory of distinct episodes. Nat Commun. 2019:10(1):203. 30643130 10.1038/s41467-018-08189-4 PMC6331638

[ref21] Ezzyat Y , Rizzuto DS. Direct brain stimulation during episodic memory. Curr Opin Biomed Eng. 2018:8:78–83.

[ref22] Ezzyat Y , Suthana N. Chapter brain stimulation. In: Oxford handbook of human memory. 2nd ed. Oxford, UK: Oxford University Press. 2024.

[ref23] Ezzyat Y , Kragel JE, Burke JF, Levy DF, Lyalenko A, Wanda PA, O’Sullivan L, Hurley KB, Busygin S, Pedisich I, et al. Direct brain stimulation modulates encoding states and memory performance in humans. Curr Biol. 2017:27(9):1251–1258. 28434860 10.1016/j.cub.2017.03.028 PMC8506915

[ref24] Ezzyat Y , Wanda P, Levy DF, Kadel A, Aka A, Pedisich I, Sperling MR, Sharan AD, Lega BC, Burks A, et al. Closed-loop stimulation of temporal cortex rescues functional networks and improves memory. Nature. Communications. 2018:9(1):365. 10.1038/s41467-017-02753-0 PMC5802791 29410414

[ref25] Fan RE , Chang KW, Hsieh CJ, Wang XR, Lin CJ. Liblinear: a library for large linear classification. J Mach Learn Res. 2008:9:1871–1874.

[ref26] Fell J , Axmacher N. The role of phase synchronization in memory processes. Nat Rev Neurosci. 2011:12(2):105–118. 21248789 10.1038/nrn2979

[ref27] Fischl B , van der Kouwe A, Destrieux C, Halgren E, Ségonne F, Salat DH, Busa E, Seidman LJ, Goldstein J, Kennedy D, et al. Automatically parcellating the human cerebral cortex. Cereb Cortex. 2004:14(1):11–22. 14654453 10.1093/cercor/bhg087

[ref28] Flöel A , Rösser N, Michka O, Knecht S, Breitenstein C. Noninvasive brain stimulation improves language learning. J Cogn Neurosci. 2008:20(8):1415–1422. 18303984 10.1162/jocn.2008.20098

[ref29] Foster DJ . Replay comes of age. Annu Rev Neurosci. 2017:40(1):581–602. 28772098 10.1146/annurev-neuro-072116-031538

[ref30] Fox MD , Buckner RL, Liu H, Chakravarty MM, Lozano AM, Pascual-Leone A. Resting-state networks link invasive and noninvasive brain stimulation across diverse psychiatric and neurological diseases. Proc Natl Acad Sci. 2014:111(41):E4367–E4375. 25267639 10.1073/pnas.1405003111 PMC4205651

[ref31] Fox KC , Shi L, Baek S, Raccah O, Foster BL, Saha S, Margulies DS, Kucyi A, Parvizi J. Intrinsic network architecture predicts the effects elicited by intracranial electrical stimulation of the human brain. Nat Hum Behav. 2020:4(10):1039–1052. 32632334 10.1038/s41562-020-0910-1 PMC7572705

[ref32] Fries P . Neuronal gamma-band synchronization as a fundamental process in cortical Computation. Annu Rev Neurosci. 2009:32(1):209–224. 19400723 10.1146/annurev.neuro.051508.135603

[ref33] Geller EB , Skarpaas TL, Gross RE, Goodman RR, Barkley GL, Bazil CW, Berg MJ, Bergey GK, Cash SS, Cole AJ, et al. Brain-responsive neurostimulation in patients with medically intractable mesial temporal lobe epilepsy. Epilepsia. 2017:58(6):994–1004. 28398014 10.1111/epi.13740

[ref34] Gramfort A , Luessi M, Larson E, Engemann DA, Strohmeier D, Brodbeck C, Parkkonen L, Hämäläinen M. MNE software for processing MEG and EEG data. NeuroImage. 2014:86:446–460. 24161808 10.1016/j.neuroimage.2013.10.027 PMC3930851

[ref35] Griffiths BJ , Martín-Buro MC, Staresina BP, Hanslmayr S. Disentangling neocortical alpha/beta and hippocampal theta/gamma oscillations in human episodic memory formation. NeuroImage. 2021:242:118454. 34358658 10.1016/j.neuroimage.2021.118454 PMC8463840

[ref36] Griffiths BJ , Parish G, Roux F, Michelmann S, van der Plas M, Kolibius LD, Chelvarajah R, Rollings DT, Sawlani V, Hamer H, et al. Directional coupling of slow and fast hippocampal gamma with neocortical alpha/beta oscillations in human episodic memory. PNAS. 2019:116(43):21834–21842. 31597741 10.1073/pnas.1914180116 PMC6815125

[ref37] Grover S , Wen W, Viswanathan V, Gill CT, Reinhart RM. Long-lasting, dissociable improvements in working memory and long-term memory in older adults with repetitive neuromodulation. Nat Neurosci. 2022:25(9):1–10. 35995877 10.1038/s41593-022-01132-3 PMC10068908

[ref38] Guderian S , Dü zel E. Induced theta oscillations mediate large-scale synchrony with mediotemporal areas during recollection in humans. Hippocampus. 2005:15(7):901–912. 16161060 10.1002/hipo.20125

[ref39] Hamani C , McAndrews M, Cohn M, Oh M, Zumsteg D, Shapiro C, Wennberg R, Lozano A. Memory enhancement induced by hypothalamic/fornix deep brain stimulation. Ann Neurol. 2008:63(1):119–123. 18232017 10.1002/ana.21295

[ref40] Hampson RE , Song D, Opris I, Santos LM, Shin DC, Gerhardt GA, Marmarelis VZ, Berger TW, Deadwyler SA. Facilitation of memory encoding in primate hippocampus by a neuroprosthesis that promotes task-specific neural firing. J Neural Eng. 2013:10(6):066013. 24216292 10.1088/1741-2560/10/6/066013 PMC3919468

[ref41] Hampson RE , Song D, Robinson BS, Fetterhoff D, Dakos AS, Roeder BM, She X, Wicks RT, Witcher MR, Couture DE, et al. Developing a hippocampal neural prosthetic to facilitate human memory encoding and recall. J Neural Eng. 2018:15(3):036014. 29589592 10.1088/1741-2552/aaaed7 PMC6576290

[ref42] Hanslmayr S , Axmacher N, Inman CS. Modulating human memory via entrainment of brain oscillations. Trends Neurosci. 2019:42(7):385–499. 10.1016/j.tins.2019.04.004 31178076

[ref43] Harris AZ , Gordon JA. Long-range neural synchrony in behavior. Annu Rev Neurosci. 2015:38(1):171–194. 25897876 10.1146/annurev-neuro-071714-034111 PMC4497851

[ref44] Hastie T , Tibshirani R, Friedman J. The elements of statistical learning. New York (NY): Springer-Verlag; 2001.

[ref45] Haufe S , Meinecke F, Görgen K, Dähne S, Haynes JD, Blankertz B, Bießmann F. On the interpretation of weight vectors of linear models in multivariate neuroimaging. NeuroImage. 2014:87:96–110. 24239590 10.1016/j.neuroimage.2013.10.067

[ref46] Hermiller MS , Chen YF, Parrish TB, Voss JL. Evidence for immediate enhancement of hippocampal memory encoding by network-targeted theta-burst stimulation during concurrent fmri. J Neurosci. 2020:40(37):7155–7168. 32817326 10.1523/JNEUROSCI.0486-20.2020 PMC7480242

[ref47] Herweg NA , Solomon EA, Kahana MJ. Theta oscillations in human memory. Trends Cogn Sci. 2020:24(3):208–227. 32029359 10.1016/j.tics.2019.12.006 PMC8310425

[ref48] Histed M , Bonin V, Reid C. Direct activation of sparse, distributed populations of cortical neurons by electrical microstimulation. Neuron. 2009:63(4):508–522. 19709632 10.1016/j.neuron.2009.07.016 PMC2874753

[ref49] Jobst BC , Kapur R, Barkley GL, Bazil CW, Berg MJ, Bergey GK, Boggs JG, Cash SS, Cole AJ, Duchowny MS, et al. Brain-responsive neurostimulation in patients with medically intractable seizures arising from eloquent and other neocortical areas. Epilepsia. 2017:58(6):1005–1014. 28387951 10.1111/epi.13739

[ref50] Kahana MJ , Ezzyat Y, Wanda PA, Solomon EA, Adamovich-Zeitlin R, Lega BC, Jobst BC, Gross RE, Ding K, Diaz-Arrastia RR. Biomarker-guided neuromodulation aids memory in traumatic brain injury. Brain Stimul. 2023:16(4):1086–1093. 37414370 10.1016/j.brs.2023.07.002

[ref51] Keerativittayayut R , Aoki R, Sarabi MT, Jimura K, Nakahara K. Large-scale network integration in the human brain tracks temporal fluctuations in memory encoding performance. elife. 2018:7:e32696. 29911970 10.7554/eLife.32696 PMC6039182

[ref52] Keller CJ , Bickel S, Entz L, Ulbert I, Milham MP, Kelly C, Mehta AD. Intrinsic functional architecture predicts electrically evoked responses in the human brain. Proc Natl Acad Sci. 2011:108(25):10308–10313. 21636787 10.1073/pnas.1019750108 PMC3121855

[ref53] Keller CJ , Huang Y, Herrero JL, Fini ME, Du V, Lado FA, Honey CJ, Mehta AD. Induction and quantification of excitability changes in human cortical networks. J Neurosci. 2018:38(23):5384–5398. 29875229 10.1523/JNEUROSCI.1088-17.2018 PMC5990984

[ref54] Khambhati AN , Kahn AE, Costantini J, Ezzyat Y, Solomon EA, Gross RE, Jobst BC, Sheth SA, Zaghloul KA, Worrell G, et al. Functional control of electrophysiological network architecture using direct neurostimulation in humans. Netw Neurosci. 2019:3(3):848–877. 31410383 10.1162/netn_a_00089 PMC6663306

[ref55] Kim H . Neural activity that predicts subsequent memory and forgetting: a meta-analysis of 74 fMRI studies. NeuroImage. 2011:54(3):2446–2461. 20869446 10.1016/j.neuroimage.2010.09.045

[ref56] Kim K , Ekstrom AD, Tandon N. A network approach for modulating memory processes via direct and indirect brain stimulation: toward a causal approach for the neural basis of memory. Neurobiol Learn Mem. 2016:134(10):162–177. 27066987 10.1016/j.nlm.2016.04.001

[ref57] Kim K , Schedlbauer A, Rollo M, Karunakaran S, Ekstrom AD, Tandon N. Network-based brain stimulation selectively impairs spatial retrieval. Brain Stimul. 2018:11(1):213–221. 29042188 10.1016/j.brs.2017.09.016 PMC5729089

[ref58] Klimesch W , Doppelmayr M, Schimke H, Ripper B. Theta synchronization and alpha desynchronization in a memory task. Psychophysiology. 1997:34(2):169–176. 9090266 10.1111/j.1469-8986.1997.tb02128.x

[ref59] Koster M , Gruber T. Rhythms of human attention and memory: an embedded process perspective. Front Hum Neurosci. 2022:16(905837). 10.3389/fnhum.2022.905837 PMC9579292 36277046

[ref60] Kragel JE , Ezzyat Y, Sperling MR, Gorniak R, Worrell GA, Berry BM, Inman C, Lin JJ, Davis KA, Das SR, et al. Similar patterns of neural activity predict memory function during encoding and retrieval. NeuroImage. 2017:155:60–71. 28377210 10.1016/j.neuroimage.2017.03.042 PMC5789770

[ref61] Kragel JE , Ezzyat Y, Worrell GA, Sperling MR, Gross RE, Lega BC, Jobst BC, Sheth SA, Zaghloul KA, Stein JM, et al. Distinct cortical systems reinstate content and context information during memory search. Nature. Communications. 2021:12(1):1–10. 10.1038/s41467-021-24393-1 PMC8295370 34290240

[ref62] Kragel JE , Schuele S, VanHaerents S, Rosenow JM, Voss JL. Rapid coordination of effective learning by the human hippocampus. Science. Advances. 2021:7(25):eabf7144. 10.1126/sciadv.abf7144 PMC8213228 34144985

[ref63] Krauss JK , Lipsman N, Aziz T, Boutet A, Brown P, Chang JW, Davidson B, Grill WM, Hariz MI, Horn A, et al. Technology of deep brain stimulation: current status and future directions. Nat Rev Neurol. 2021:17(2):75–87. 33244188 10.1038/s41582-020-00426-z PMC7116699

[ref64] Kucewicz MT , Berry B, Miller L, Khadjevand F, Ezzyat Y, Stein J, Kremen V, Brinkman B, Wanda P, Sperling M, et al. Evidence for verbal memory enhancement with electrical brain stimulation in the lateral temporal cortex. Brain. 2018:141(4):971–978. 29324988 10.1093/brain/awx373

[ref65] Lee H , Fell J, Axmacher N. Electrical engram: how deep brain stimulation affects memory. Trends Cogn Sci. 2013:17(11):574–584. 24126128 10.1016/j.tics.2013.09.002

[ref66] Limousin P , Krack P, Pollak P, Benazzouz A, Ardouin C, Hoffmann D, Benabid AL. Electrical stimulation of the subthalamic nucleus in advanced Parkinson’s disease. N Engl J Med. 1998:339(16):1105–1111. 9770557 10.1056/NEJM199810153391603

[ref67] Long NM , Burke JF, Kahana MJ. Subsequent memory effect in intracranial and scalp EEG. Neuroimage. 2014:84:488–494. 24012858 10.1016/j.neuroimage.2013.08.052 PMC3849113

[ref68] Lozano AM , Lipsman N. Probing and regulating dysfunctional circuits using deep brain stimulation. Neuron. 2013:77(3):406–424. 23395370 10.1016/j.neuron.2013.01.020

[ref69] Lozano AM , Fosdick L, Chakravarty MM, Leoutsakos JM, Munro C, Oh E, Drake KE, Lyman CH, Rosenberg PB, Anderson WS, et al. A phase ii study of fornix deep brain stimulation in mild alzheimer’s disease. J Alzheimers Dis. 2016:54(2):777–787. 27567810 10.3233/JAD-160017 PMC5026133

[ref70] Lujan JL , Chaturvedi A, Choi KS, Holtzheimer PE, Gross RE, Mayberg HS, McIntyre CC. Tractography-activation models applied to subcallosal cingulate deep brain stimulation. Brain Stimul. 2013:6(5):737–739. 23602025 10.1016/j.brs.2013.03.008 PMC3772993

[ref71] Mankin EA , Fried I. Modulation of human memory by deep brain stimulation of the entorhinal-hippocampal circuitry. Neuron. 2020:106(2):218–235. 32325058 10.1016/j.neuron.2020.02.024 PMC7347298

[ref72] Mankin EA , Aghajan ZM, Schuette P, Tran ME, Tchemodanov N, Titiz A, Kalender G, Eliashiv D, Stern J, Weiss SA, et al. Stimulation of the right entorhinal white matter enhances visual memory encoding in humans. Brain Stimul. 2021:14(1):131–140. 33279717 10.1016/j.brs.2020.11.015 PMC7855810

[ref73] Mayberg H , Lozano A, Voon V, McNeely H, Seminowicz D, Hamani C, Schwalb J, Kennedy S. Deep brain stimulation surgery for treatment resistant depression. Neuron. 2005:45(5):651–660. 15748841 10.1016/j.neuron.2005.02.014

[ref74] McIntyre CC , Hahn PJ. Network perspectives on the mechanisms of deep brain stimulation. Neurobiol Dis. 2010:38(3):329–337. 19804831 10.1016/j.nbd.2009.09.022 PMC2862840

[ref75] Mohan UR , Watrous AJ, Miller JF, Lega BC, Sperling MR, Worrell GA, Gross RE, Zaghloul KA, Jobst BC, Davis KA, et al. The effects of direct brain stimulation in humans depend on frequency, amplitude, and white-matter proximity. Brain Stimul. 2020:13(5):1183–1195. 32446925 10.1016/j.brs.2020.05.009 PMC7494653

[ref76] Moriarity J , Boatman D, Krauss G, Storm P, Lenz F. Human "memories" can be evoked by stimulation of the lateral temporal cortex after ipsilateral medial temporal lobe resection. J Neurol Neurosurg Psychiatry. 2001:71(4):549–551. 11561047 10.1136/jnnp.71.4.549 PMC1763507

[ref77] Morrell MJ . Responsive cortical stimulation for the treatment of medically intractable partial epilepsy. Neurology. 2011:77(13):1295–1304. 21917777 10.1212/WNL.0b013e3182302056

[ref78] Nilakantan AS , Bridge DJ, Gagnon EP, VanHaerents SA, Voss JL. Stimulation of the posterior cortical-hippocampal network enhances precision of memory recollection. Curr Biol. 2017:27(3):465–470. 28111154 10.1016/j.cub.2016.12.042 PMC5302852

[ref79] Nowak L , Bullier J. Axons, but not cell bodies, are activated by electrical stimulation in cortical gray matter i. evidence from chronaxie measurements: I. Evidence from chronaxie measurements. Exp Brain Res. 1998:118(4):477–488. 9504843 10.1007/s002210050304

[ref80] Ojemann GA , Creutzfeldt O, Lettich E, Haglund M. Neuronal activity in human lateral temporal cortex related to short-term verbal memory, naming and reading. Brain. 1988:111(6):1383–1403. 3208062 10.1093/brain/111.6.1383

[ref81] Osipova D , Takashima A, Oostenveld R, Fernández G, Maris E, Jensen O. Theta and gamma oscillations predict encoding and retrieval of declarative memory. J Neurosci. 2006:26(28):7523–7531. 16837600 10.1523/JNEUROSCI.1948-06.2006 PMC6674196

[ref82] Paulk AC , Zelmann R, Crocker B, Widge AS, Dougherty DD, Eskandar EN, Weisholtz DS, Richardson RM, Cosgrove GR, Williams ZM, et al. Local and distant cortical responses to single pulse intracranial stimulation in the human brain are differentially modulated by specific stimulation parameters. Brain Stimul. 2022:15(2):491–508. 35247646 10.1016/j.brs.2022.02.017 PMC8985164

[ref83] Pedregosa F , Varoquaux G, Gramfort A, Michel V, Thirion B, Grisel O, Blondel M, Prettenhofer P, Weiss R, Dubourg V, et al. Scikit-learn: machine learning in python. J Mach Learn Res. 2011:12:2825–2830.

[ref84] Perrine K , Devinsky O, Uysal S, Luciano D, Dogali M. Left temporal neocortex mediation of verbal memory: evidence from functional mapping with cortical stimulation. Neurology. 1994:44(10):1845–1850. 7936234 10.1212/wnl.44.10.1845

[ref85] Reinhart RM , Nguyen JA. Working memory revived in older adults by synchronizing rhythmic brain circuits. Nat Neurosci. 2019:22(5):820–827. 30962628 10.1038/s41593-019-0371-x PMC6486414

[ref86] Reinhart RM , Cosman JD, Fukuda K, Woodman GF. Using transcranial direct-current stimulation (tdcs) to understand cognitive processing. Atten Percept Psychophys. 2017:79(1):3–23. 27804033 10.3758/s13414-016-1224-2 PMC5539401

[ref87] Sankar T , Lipsman N, Lozano AM. Deep brain stimulation for disorders of memory and cognition. Neurotherapeutics. 2014:11(3):527–534. 24777384 10.1007/s13311-014-0275-0 PMC4121440

[ref88] Scangos KW , Khambhati AN, Daly PM, Makhoul GS, Sugrue LP, Zamanian H, Liu TX, Rao VR, Sellers KK, Dawes HE, et al. Closed-loop neuromodulation in an individual with treatment-resistant depression. Nat Med. 2021:27(10):1696–1700. 34608328 10.1038/s41591-021-01480-w PMC11219029

[ref89] Scangos KW , Makhoul GS, Sugrue LP, Chang EF, Krystal AD. State-dependent responses to intracranial brain stimulation in a patient with depression. Nat Med. 2021:27(2):229–231. 33462446 10.1038/s41591-020-01175-8 PMC8284979

[ref90] Shannon RV . A model of safe levels for electrical stimulation. IEEE Trans Biomed Eng. 1992:39(4):424–426. 1592409 10.1109/10.126616

[ref91] Siddiqi SH , Kording KP, Parvizi J, Fox MD. Causal mapping of human brain function. Nat Rev Neurosci. 2022:23(6):361–375. 35444305 10.1038/s41583-022-00583-8 PMC9387758

[ref92] Solomon EA , Kragel JE, Sperling MR, Sharan AD, Worrell GA, Kucewicz MT, Inman CS, Lega BC, Davis KA, Stein JM, et al. Widespread theta synchrony and high-frequency desynchronization underlies enhanced cognition. Nat Commun. 2017:8(1):1704. 29167419 10.1038/s41467-017-01763-2 PMC5700170

[ref93] Solomon EA , Kragel JE, Gross RE, Lega BC, Michael SG, Worrell R, Sheth SA, Zaghloul KA, Jobst BC, Stein JM, et al. Medial temporal lobe functional connectivity predicts stimulation-induced theta power. Nature. Communications. 2018:9(1):4437. 10.1038/s41467-018-06876-w PMC6202342 30361627

[ref94] Solomon EA , Sperling MR, Sharan AD, Wanda PA, Levy DF, Lyalenko A, Pedisich I, Rizzuto DS, Kahana MJ. Theta-burst stimulation entrains frequency-specific oscillatory responses. Brain Stimul. 2021:14(5):1271–1284. 34428553 10.1016/j.brs.2021.08.014 PMC9161680

[ref95] Sreekumar V , Witting JH Jr, Sheehan TC, Zaghloul KA. Principled approaches to direct brain stimulation for cognitive enhancement. Front Neurosci. 2017:11:1–7. 10.3389/fnins.2017.00650 PMC5714894 29249927

[ref96] Staresina BP , Wimber M. A neural chronometry of memory recall. Trends Cogn Sci. 2019:23(12):1071–1085. 31672429 10.1016/j.tics.2019.09.011

[ref97] Staudigl T , Hanslmayr S. Theta oscillations at encoding mediate the context-dependent nature of human episodic memory. Curr Biol. 2013:23(12):1101–1106. 23746635 10.1016/j.cub.2013.04.074

[ref98] Stiso J , Khambhati AN, Menara T, Kahn AE, Stein JM, Das SR, Gorniak R, Tracy J, Litt B, Davis KA, et al. White matter network architecture guides direct electrical stimulation through optimal state transitions. Cell Rep. 2019:28(10):2554–2566. 31484068 10.1016/j.celrep.2019.08.008 PMC6849479

[ref99] Sun FT , Morrell MJ, Wharen RE. Responsive cortical stimulation for the treatment of epilepsy. Neurotherapeutics. 2008:5(1):68–74. 18164485 10.1016/j.nurt.2007.10.069 PMC5084128

[ref100] Suthana N , Fried I. Deep brain stimulation for enhancement of learning and memory. NeuroImage. 2014:85(0 3):996–1002. 23921099 10.1016/j.neuroimage.2013.07.066 PMC4445933

[ref101] Suthana N , Haneef Z, Stern J, Mukamel R, Behnke E, Knowlton B, Fried I. Memory enhancement and deep-brain stimulation of the entorhinal area. N Engl J Med. 2012:366(6):502–510. 22316444 10.1056/NEJMoa1107212 PMC3447081

[ref102] Tambini A , Nee DE, D’Esposito M. Hippocampal-targeted theta-burst stimulation enhances associative memory formation. J Cogn Neurosci. 2018:30(10):1452–1472. 29916791 10.1162/jocn_a_01300 PMC7467684

[ref103] Ter Wal M , Linde-Domingo J, Lifanov J, Roux F, Kolibius LD, Gollwitzer S, Lang J, Hamer H, Rollings D, Sawlani V, et al. Theta rhythmicity governs human behavior and hippocampal signals during memory-dependent tasks. Nat Commun. 2021:12(1):1–15. 34857748 10.1038/s41467-021-27323-3 PMC8639755

[ref104] Titiz AS , Hill MR, Mankin EA, Aghajan ZM, Eliashiv D, Tchemodanov N, Maoz U, Stern J, Tran ME, Schuette P, et al. Theta-burst microstimulation in the human entorhinal area improves memory specificity. elife. 2017:6:e29515. 10.7554/eLife.29515 PMC5655155 29063831

[ref105] Voytek B , Knight RT. Dynamic network communication as a unifying neural basis for cognition, development, aging, and disease. Biol Psychiatry. 2015:77(12):1089–1097. 26005114 10.1016/j.biopsych.2015.04.016 PMC4443259

[ref106] Warren KN , Hermiller MS, Nilakantan AS, Voss JL. Stimulating the hippocampal posterior-medial network enhances task-dependent connectivity and memory. elife. 2019:8:e49458. 31724946 10.7554/eLife.49458 PMC6855798

[ref107] Watrous AJ , Tandon N, Conner CR, Pieters T, Ekstrom AD. Frequency-specific network connectivity increases underlie accurate spatiotemporal memory retrieval. Nat Neurosci. 2013:16(3):349–356. 23354333 10.1038/nn.3315 PMC3581758

[ref108] Wixted JT . Dual-process theory and signal-detection theory of recognition memory. Psychol Rev. 2007:114(1):152–176. 17227185 10.1037/0033-295X.114.1.152

[ref109] Yushkevich PA , Pluta JB, Wang H, Xie L, Ding SL, Gertje EC, Mancuso L, Kliot D, Das SR, Wolk DA. Automated volumetry and regional thickness analysis of hippocampal subfields and medial temporal cortical structures in mild cognitive impairment. Hum Brain Mapp. 2015:36(1):258–287. 25181316 10.1002/hbm.22627 PMC4313574

